# Supplementary material for: An intronic copy number variation in Syntaxin 17 determines speed of greying and melanoma incidence in Grey horses
Source: Nat Commun. 2024 Aug 29;15:7510. doi: 10.1038/s41467-024-51898-2 (PMC11362437; doi:10.1038/s41467-024-51898-2)
Supplement: Supplementary file 1 — Supplementary Information [file 41467_2024_51898_MOESM1_ESM.pdf]

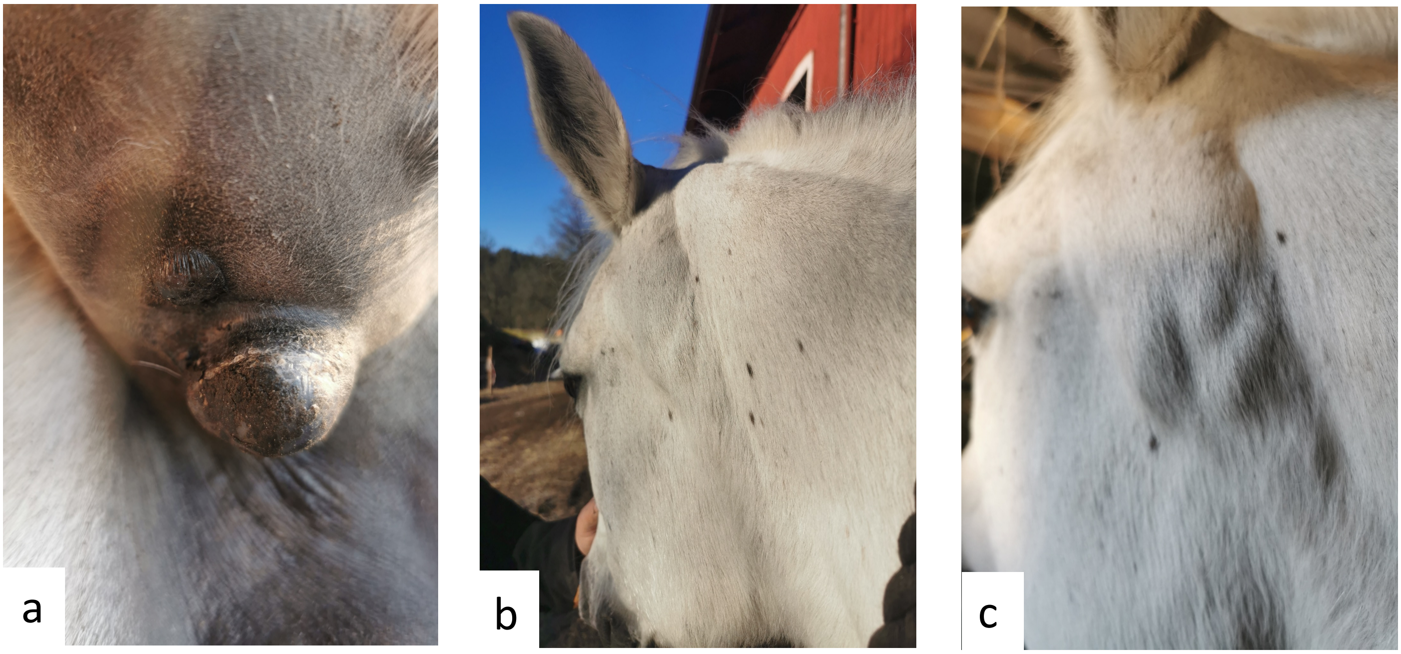

Supplementary Fig. 1. Melanoma in grey Connemara Ponies. The most common locations of visual melanoma in Grey horses are under the tail and in the jaw angel (parotis area).
(a) Prominent melanoma under the tail of a 12 years old Connemara mare. The father is Hagens D'Arcy and the mother is an unidentified fast greying horse. **(b)** Melanoma in the jaw angle accumulated around the lymphatic tissue (most likely metastasis) of a 15 years old Connemara mare. The father is Hagens D'Arcy and the mother is a random fast greying mare. **(c)** A zoomed in view of Supplementary Fig. 1b where the lymphatic tissue-associated melanoma visually bulges under the skin. The two melanoma displaying mares were not part of the mapping pedigree since their mothers were fast greying horses. Photo: Elisabeth Ljungstorp.

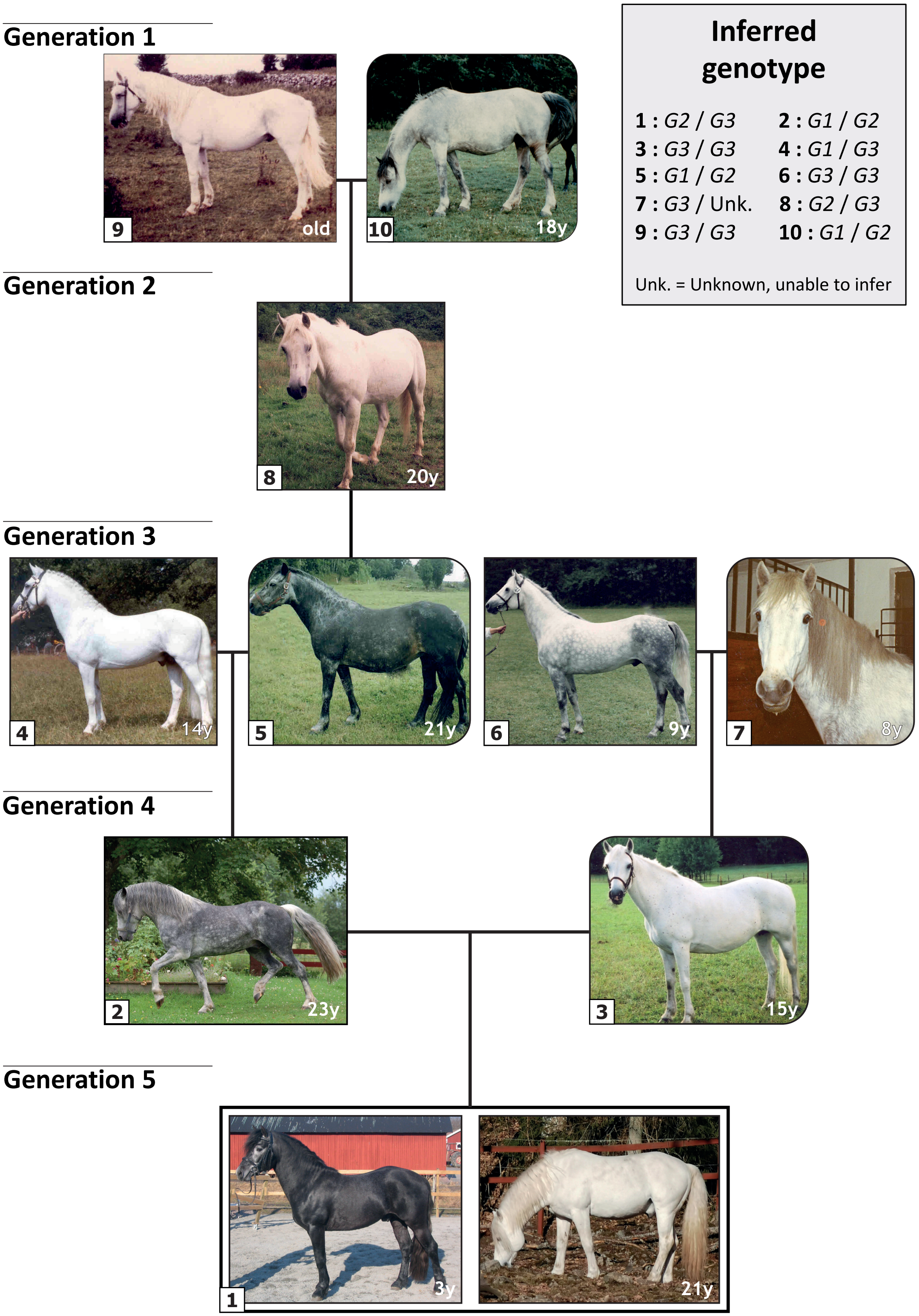

Supplementary Fig. 2. Five generation pedigree of Connemara Ponies segregating for slow and fast greying. The sire in generation 5 (Hagens D'Arcy) was postulated to be heterozygous for slow/fast greying because his parents were fast greying (mother) and slow greying (father) and in matings with non-grey dams he had progeny that were either classified as slow or fast greying. The inferred genotypes of all horses in the pedigree are based on information listed in Supplementary Table 6. Photo: 1 Elisabeth Ljungstorp, 2 Marlen Näslin, 3 Madeleine Beckman, 4 Anneli Dahlskog, 5-8 Madeleine Beckman, 9 Beckman Archive, 10 Madeleine Beckman.

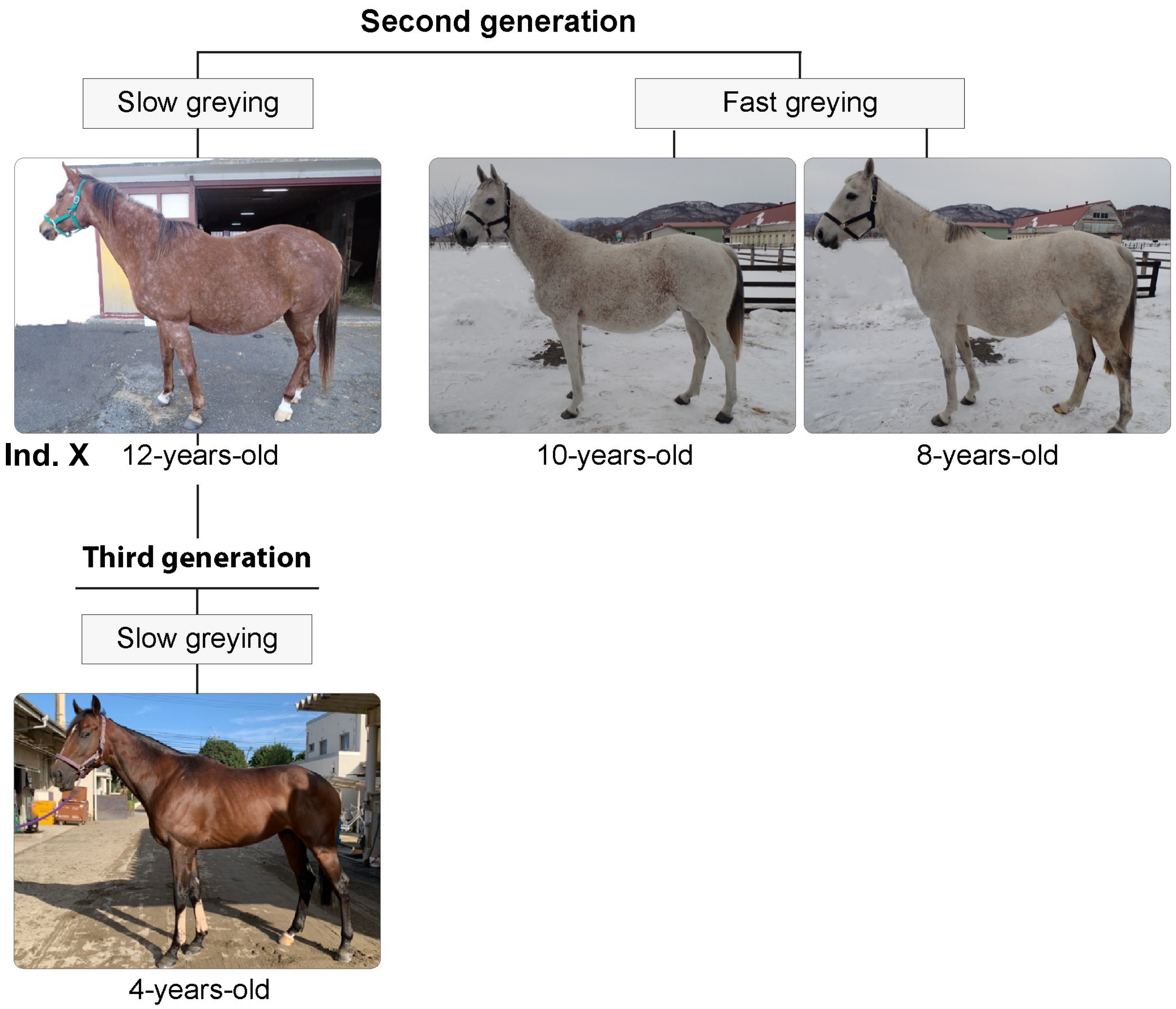

Supplementary Fig. 3. Three generation pedigree of Japanese Thoroughbred horses segregating for fast and slow greying. The dam in the first generation (not shown) was fast greying according to the Japanese Thoroughbred Stud Book. She produced one slow greying (Individual X) and two fast greying progenies in matings with non-grey sires (second generation). Three slow greying horses from the third generation were offspring of the slow greying dam (individual X in generation two) in matings with non-grey sires. One of these horses is included here. Photos were taken by Hajime Ohmura and Kazuhiro Seki.

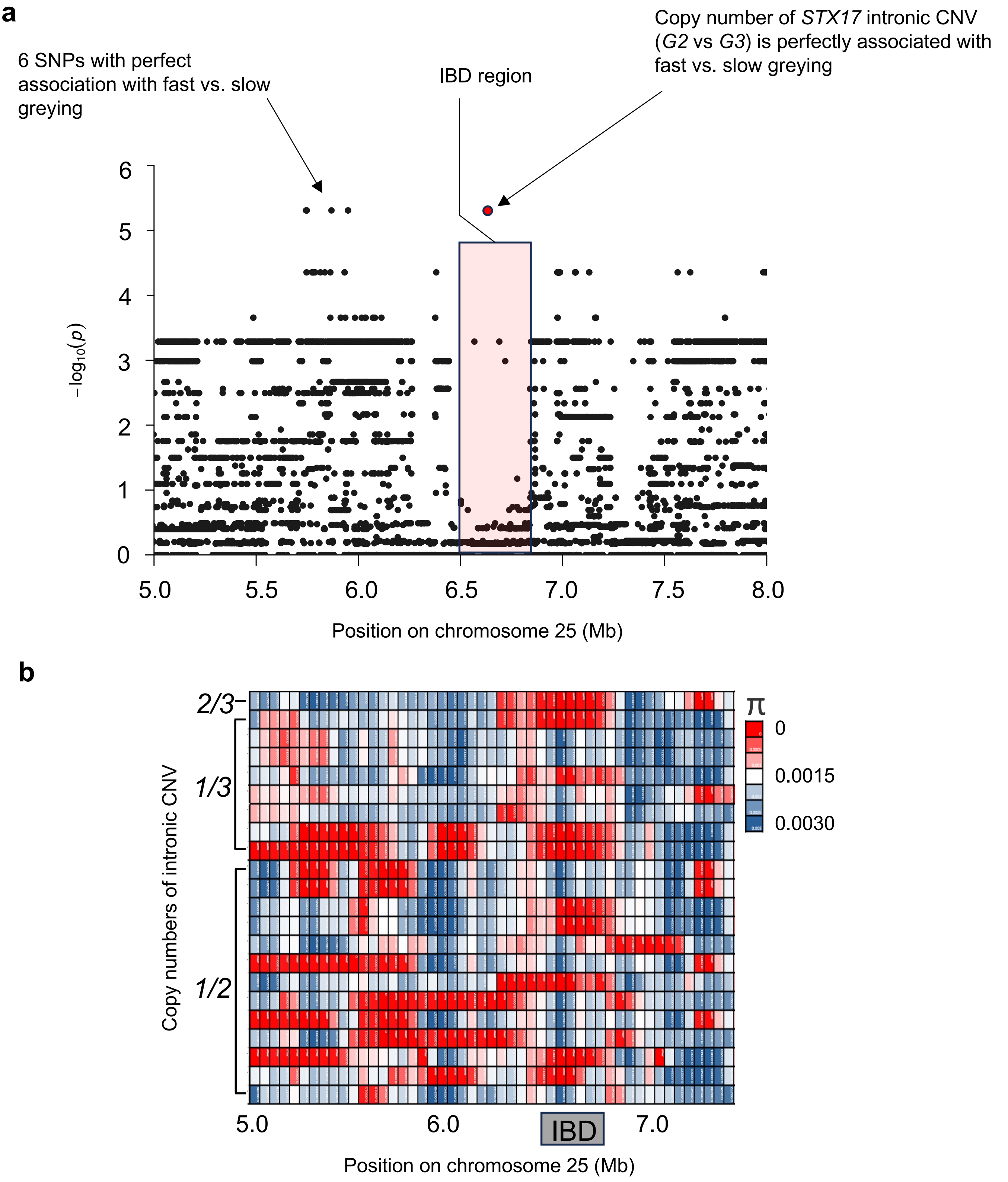

Supplementary Fig. 4. GWAS analysis. (a) GWAS results for the region chr25:5-8 Mb comparing 8 fast greying individuals with 13 slow greying individuals using single SNPs. The IBD region (chr25:6,508,360-6,860,309 bp) is indicated by the red box. A clear reduction in the number of informative SNPs is noted within the IBD-region, where *STX17* CNV genotypes (*G2* vs. *G3*) are perfectly associated with slow vs. fast greying (red circle). (**b**) Nucleotide diversity (π) in windows of 100 kb in the region flanking *STX17*. Deduced *Grey* genotypes are indicated to the left. The sire Hagen's D'Arcy is row 1 and shows homozygosity for the 350 kb IBD region shared by all *G2* and *G3* haplotypes.

a

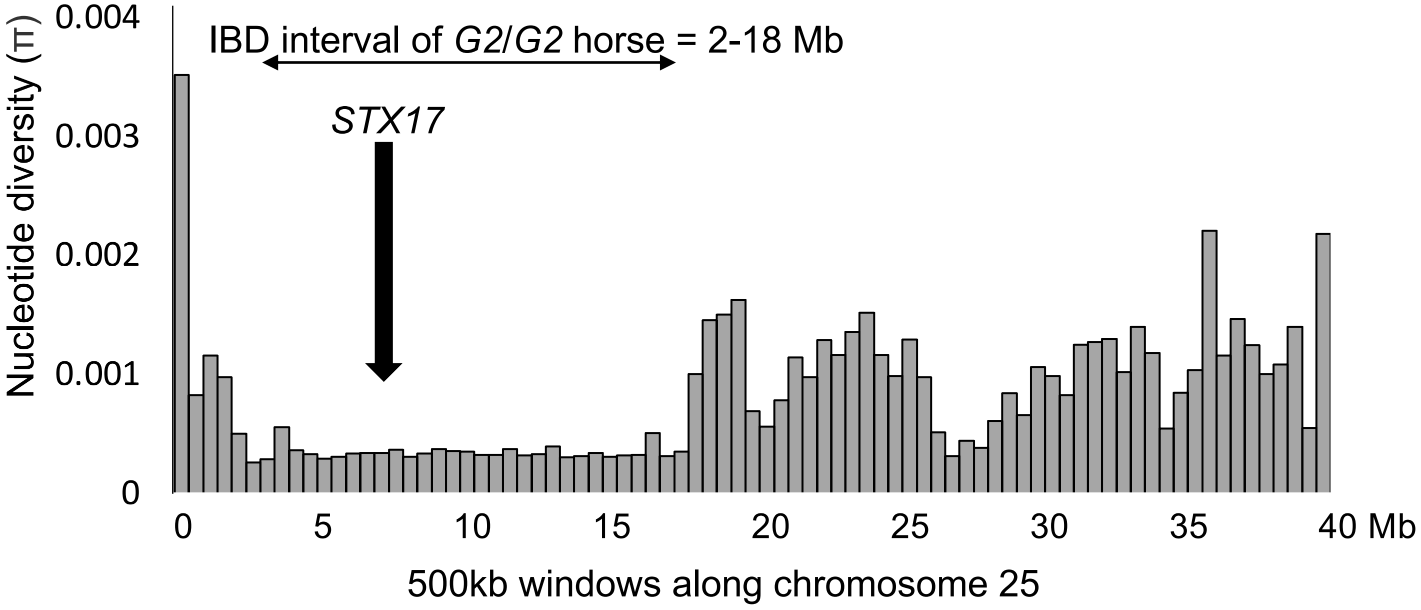

b

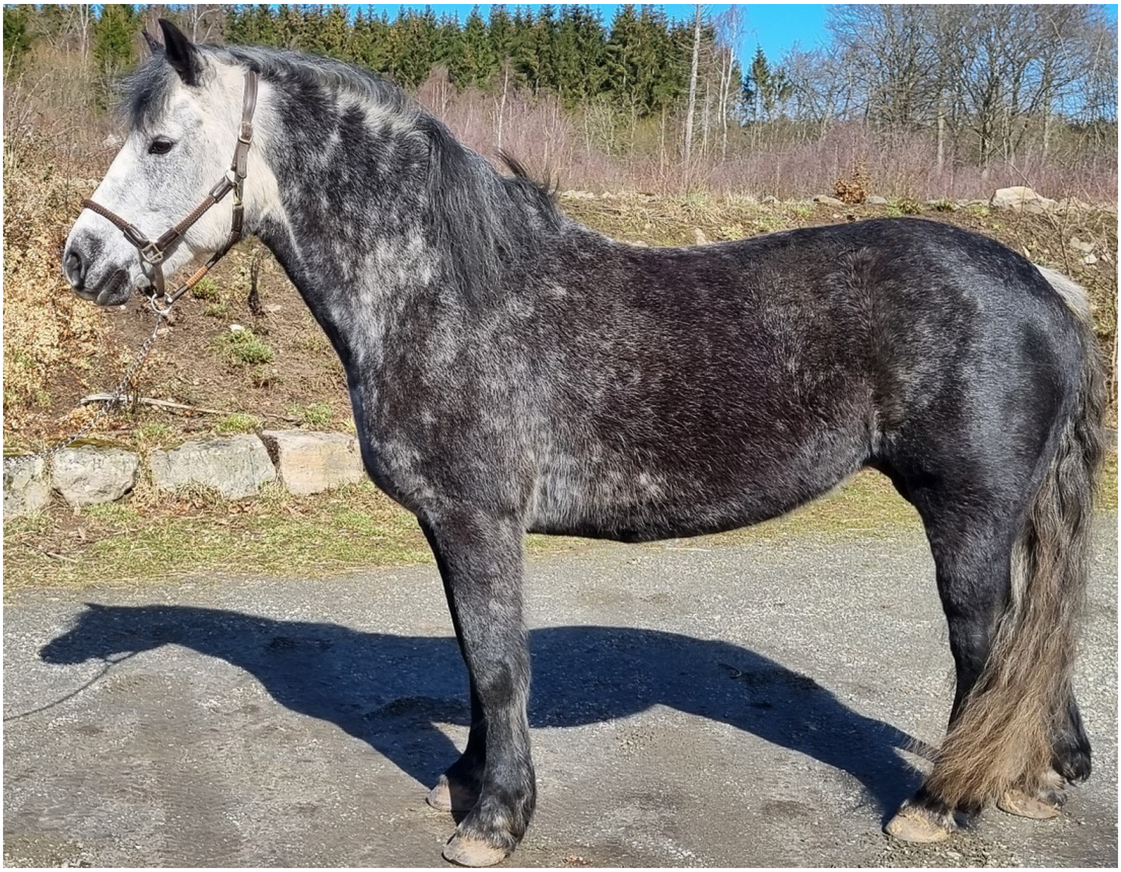

Supplementary Fig. 5. Runs of homozygosity and greying phenotype of a *G2/G2* homozygote from the Connemara pony breed. (a) Nucleotide diversity (π) estimated from Oxford nanopore sequencing data along chromosome 25. Average π values for 500 kb windows are shown. **(b)** Phenotype at the age of 11 years. Photo: Madeleine Beckman.

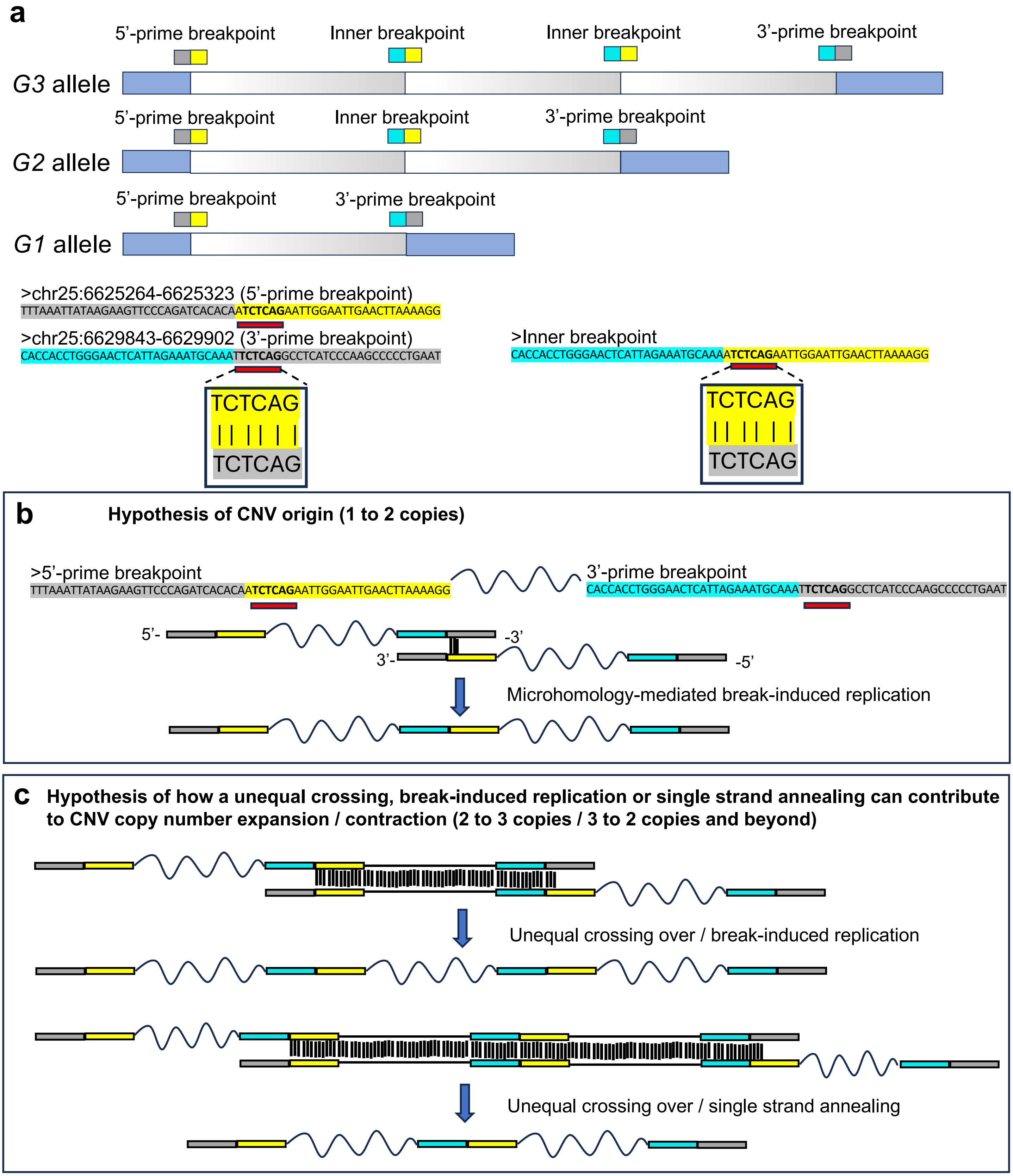

Supplementary Fig. 6. Duplication breakpoint analysis for *STX17*.
(a) CNV breakpoint sequences for consensus sequences of *G2* and *G3* alleles based on Oxford nanopore sequencing of the Connemara *G2/G3* sire and corresponding reference sequences for the *G1* haplotype (equCab3 reference genome). A 6-base sequence motif showing micro-homology between the 5'- and 3'-breakpoint sequences is high-lighted. (**b**) The 6-base motif showing sequence homology may have triggered micro-homology break-induced replication, creating a duplication allele (*G2*) in the germline. (**c**) Once duplicated, further copy number expansions/retractions of the CNV may involve DNA double-strand break repair pathways such as Non Allelic Homologous Replication (NAHR) or single strand annealing.

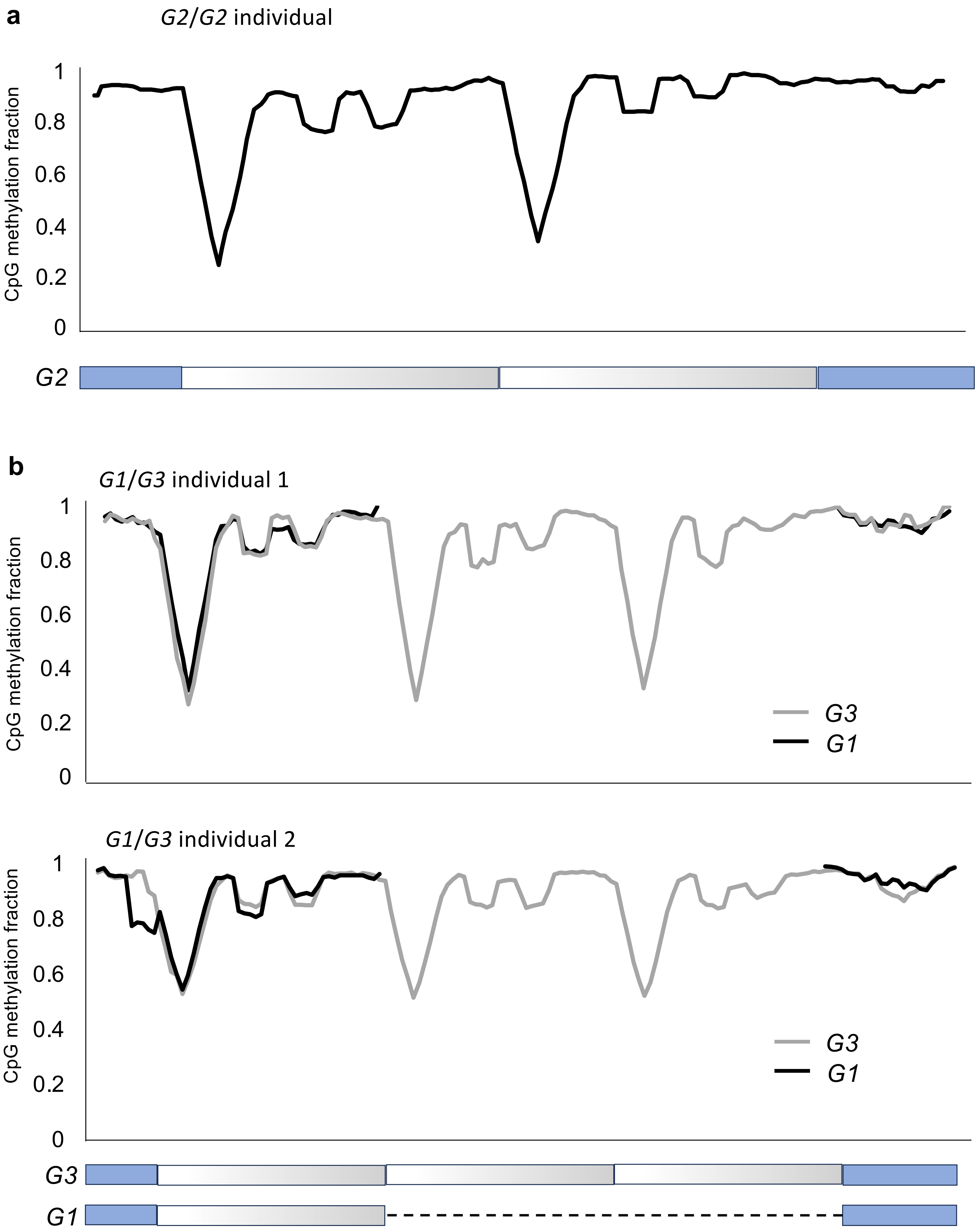

Supplementary Fig. 7. CpG methylation frequency in blood DNA. Data for the Connemara *G2/G2* homozygote (**a**) and two *G1/G3* heterozygous Quarter horses (**b**) are presented. Oxford nanopore sequence reads were used to infer average CpG methylation levels in sliding windows of 5 CpG sites along the part of *STX17* intron 6 showing copy number variation. The nanopore reads used in this analysis spanned the entire region.

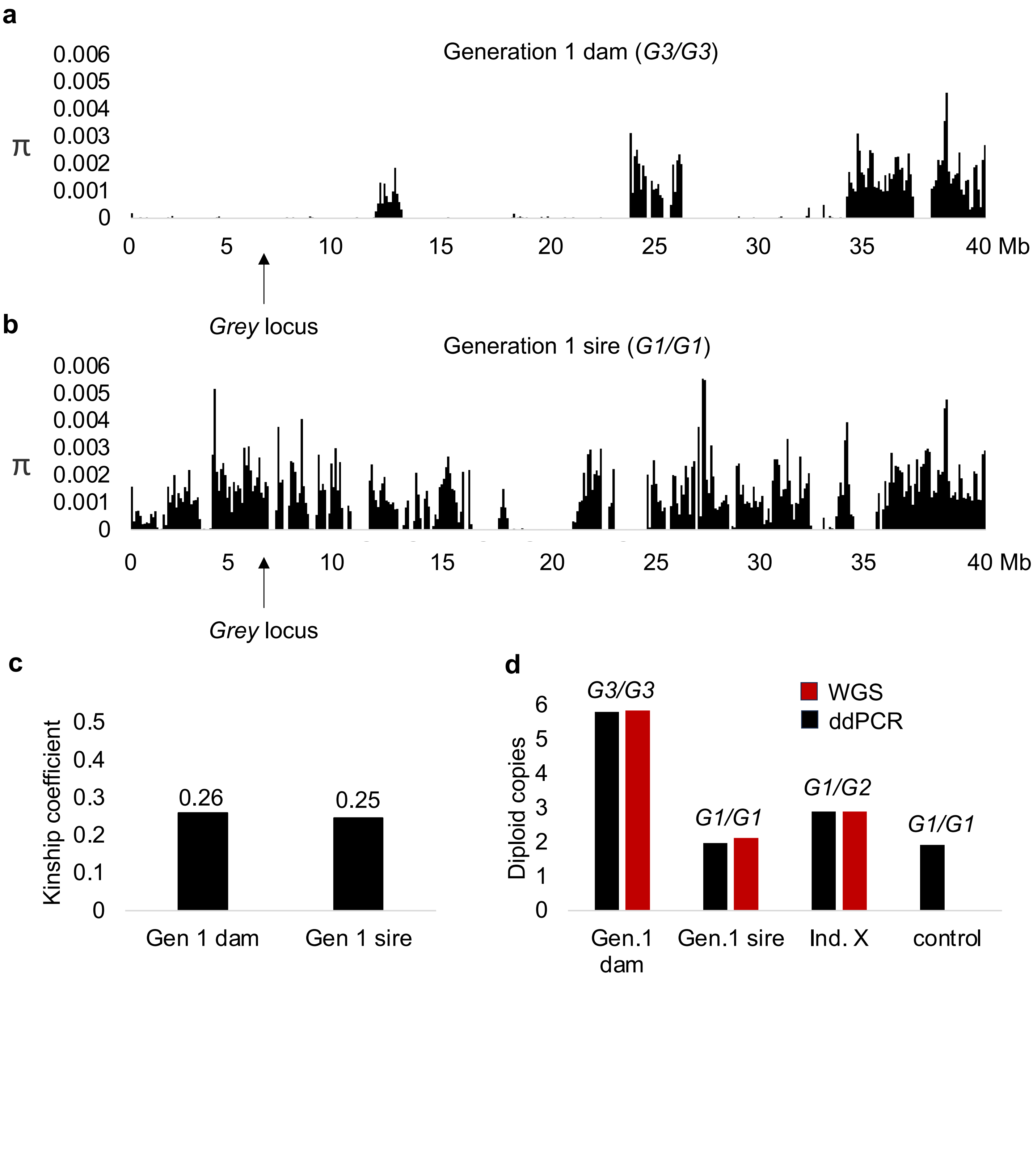

Supplementary Fig. 8. Characterization of the Japanese Thoroughbred trio. Nucleotide diversity along chromosome 25 for (**a**) the dam and (**b**) the sire in generation 1 (**Figure 5**). (**c**) Kinship coefficients based on 12.18 million SNPs between Individual X (*G1/G2*) and the Generation 1 dam (*G3/G3*) and sire (*G1/G1*), showing that Individual X is their offspring. (**d**) Duplication copy number estimates based on Digital droplet PCR (ddPCR) and Whole Genome Sequencing (WGS) for the *STX17* CNV in the trio and in a control horse without duplication (only ddPCR).

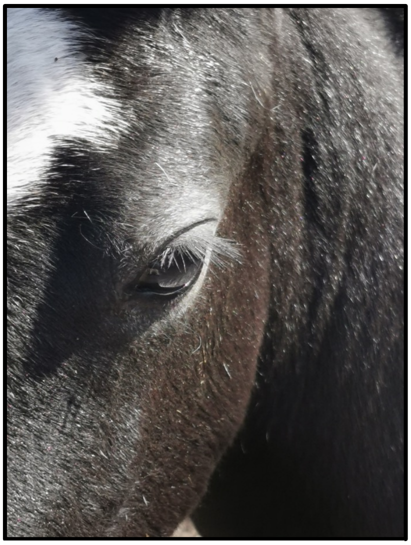

a) Fast grey, 2 week old

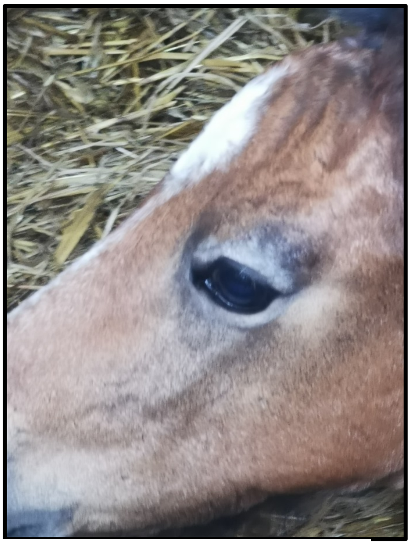

b) Fast grey, 1 month old

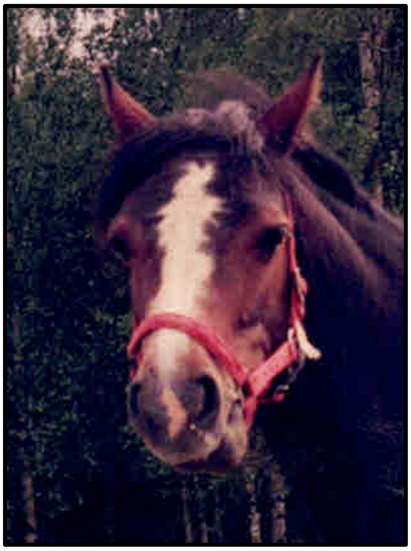

c) Slow grey, 5 years old

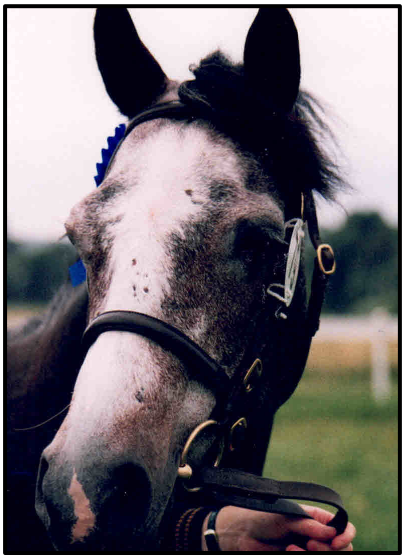

d) Slow grey, 11 years old

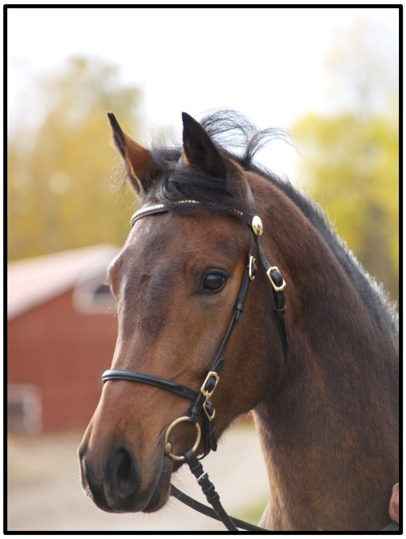

e) Slow grey, 2 years old

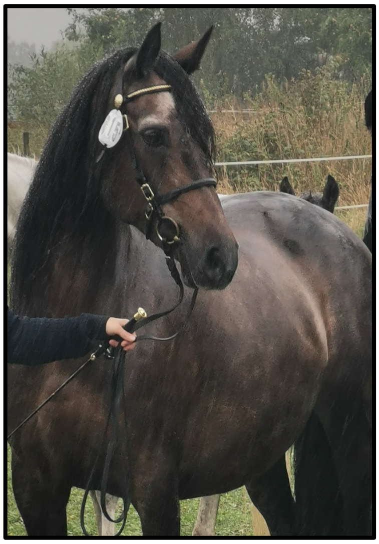

f) Slow grey, 12 years old

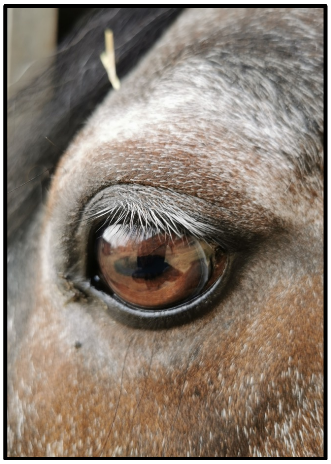

g) Slow grey, 13 years old

Supplementary Fig. 9. Comparison of the coat color phenotypes of fast and slow greying Connemara ponies from younger age and onwards. (a) Fast greying foal 2 weeks old. Grey hairs visible on the eyelids. (**b**) Fast greying foal about 1 month old. Circles of grey hairs around the eyes. (**c, d**) Slow greying mare at 5 and 11 years, showing a white blaze with shattering edges, slowly spreading out. (**e, f, g**) Slow greying mare at 2, 12, and 13 years. Pure bay with no visible grey hairs at 2 years of age. Eyelashes turning grey between 12 and 13 years of age. Photo: Elisabeth Ljungstorp (**a, b, e, f, g**) and Madeleine Beckman (**c, d**).

Supplementary Table 1. Inferred *Grey* genotypes for the horses included in Supplementary Fig. 2.

Horse	Genotype	Comments
1	*G2/G3*	Only fast or slow greying foals from 25 matings to non-grey mares
2	*G1/G2*	Only slow greying or non-grey foals from 33 matings to non-grey mares
3	*G3/G3*	10 foals, all fast greys, 8 of them by non-grey stallions
4	*G1/G3*	Fast greying individual with non-grey sire
5	*G1/G2*	Slow greying individual with 16 foals, 4 of them where non-grey
6	*G3/G3*	87 registered foals, all fast greys
7	*G3*/Unk.	17 foals all fast grey, 9 of them by non-grey stallions but no clear phenotype data available on 4 of them
8	*G2/G3*	Sire *G3/G3*, Dam *G2/G1*. Approximately 70 foals registered in Ireland, both fast and slow greying individuals
9	*G3/G3*	Approximately 300 foals in Ireland, very few non-greys registered (not parentage tested). 31 foals registered in Sweden, all fast greys.
10	*G1/G2*	Slow greying individual with non-grey sire

Unk. = unknown. Not possible to infer the genotype.

Supplementary Table 2. Individuals from the Connemara pony and Japanese Thoroughbred pedigrees subjected to whole genome sequencing (WGS). The Connemara sire, Hagens D'Arcy (HD) was not included in the GWAS analysis. Inferred copy numbers from WGS depths of coverage is shown alongside copy numbers inferred from ddPCR data. GWAS encoding is the phenotype used in the PLINK analysis: 1=Fast Grey, 2=Slow Grey.

				**Estimated copy number**	
**Horse ID**	**Generation**	**Grey phenotype**	**GWAS encoding**	**ddPCR**	**WGS**
Connemara ponies					
Hagens D'Arcy	G0	Fast	Not used	4.75	4.5
HD_offspring 1	G1	Fast	1	3.77	3.8
HD_offspring 2	G1	Slow	2	2.92	2.6
HD_offspring 3	G1	Slow	2	3.03	2.8
HD_offspring 4	G1	Fast	1	3.82	3.7
HD_offspring 11	G1	Slow	2	2.99	2.5
HD_offspring 6	G1	Fast	1	3.78	3.6
HD_offspring 7	G1	Slow	2	2.84	2.9
HD_offspring 8	G1	Slow	2	2.93	2.5
HD_offspring 9	G1	Slow	2	2.87	3.1
HD_offspring 10	G1	Slow	2	2.94	2.9
HD_offspring 5	G1	Slow	2	2.77	2.9
HD_offspring 12	G1	Fast	1	3.82	3.8
HD_offspring 13	G1	Slow	2	2.89	3.2
HD_offspring 14	G1	Slow	2	3.10	2.8
HD_offspring 15	G1	Fast	1	3.93	3.3
HD_offspring 16	G1	Fast	1	4.03	4.0
Japanese Thoroughbred					
2021-06	G1	Slow	2	2.92	2.3
2021-07	G2	Slow	2	2.95	3.1
2022-04	G2	Slow	2	2.89	2.9
2022-05	G1	Fast	1	3.82	4.1
2022-06	G1	Fast	1	3.85	3.8

Supplementary Table 3. Location and closest genes for six SNPs on horse chromosome 25 with perfect association with speed of greying. PhyloP scores were derived from alignments of 100 Vertebrate genomes and were accessed from the University of California Santa Cruz (UCSC) genome browser website (https://genome-euro.ucsc.edu/).

SNP position	Closest gene	PhyloP*
5,743,983	Intron of *COL15A1*	0.21
5,747,798	Intron of *COL15A1*	-0.80
5,747,815	Intron of *COL15A1*	-0.67
5,868,699	Intergenic *COL15A1* / *TGFBR1*	NA*
5,949,891	Intergenic *TGFBR1* / *ALG2*	-0.02
5,949,910	Intergenic *TGFBR1* / *ALG2*	0.22

*NA: No PhyloP conservation score was available for this position

Supplementary Table 4. Age distribution of 25 slow greying (*G1/G2*) Connemara ponies examined for the incidence of melanoma. None of the horses was diagnosed with melanoma.

Horse	Age at observation
1	35
2	33
3	21
4	15
5	18
6	19
7	22
8	17
9	16
10	15
11	16
12	21
13	16
14	17
15	17
16	22
17	22
18	27
19	28
20	18
21	22
22	15
23	22
24	17
25	15

Supplementary Table 5. **Guide RNAs used for Cas9 sequence capture of the *STX17* duplicated region on horse chromosome 25.**

**Coordinates (EquCab3)**	**Sequence**	**Strand**
6,623,878-6,623,900	ATCCTGGGAAACCTTAGAAG	+
6,623,747-6,623,769	GCAAATAAACTCTAAACATG	+
6,631,636-6,631,658	TCACAGCAGCAGTGTTGACA	-
6,631,564-6,631,586	TCACAGCAGCAGTGTTGACA	-
