# Supplementary material for: An intronic copy number variation in Syntaxin 17 determines speed of greying and melanoma incidence in Grey horses
Source: Nat Commun. 2024 Aug 29;15:7510. doi: 10.1038/s41467-024-51898-2 (PMC11362437; doi:10.1038/s41467-024-51898-2)
Supplement: Supplementary file 3 — Description of Additional Supplementary Files [file 41467_2024_51898_MOESM3_ESM.pdf]

Description of Additional Supplementary Files

File Name: Supplementary Data 1

Description: Copy number variation for the 4.6 kb duplication in STX17 intron 6 in 1,400 horses across 78 populations. Data based on genotyping services provided at the UC Davis Veterinary Genetics Laboratory.

File Name: Supplementary Data 2

Description: Individual copy number data for grey duplication/triplication based on ddPCR methodology using two test assays, Dup A and Dup B, as well as a reference assay. Dup A was designed within the grey CNV and indicates the total number of copies present. DupB is designed across the duplication junction - it is not expected to amplify in wild-type (non-grey) animals and gives supporting information for the interpretation of Dup A. The reference assay was designed for a control gene expected to have two copies. The ratio of Dup A and Dup B presented are calculated based on the reference assay. Poisson Ratio Max and Poisson Ratio Min are also presented indicating the Poisson confidence limits for each of the test assays.
